# Supplementary material for: Controlling network ensembles
Source: Nat Commun. 2021 Mar 25;12:1884. doi: 10.1038/s41467-021-22172-6 (PMC7994643; doi:10.1038/s41467-021-22172-6)
Supplement: Supplementary file 1 — Supplementary Information [file 41467_2021_22172_MOESM1_ESM.pdf]

Controlling Network Ensembles
Supplementary Information

Isaac Klickstein and Francesco Sorrentino

February 11, 2021

Supplementary Note 1: Derivations of Results

Solution of the Optimal Control Problem

In the main text, we are interested in solving the following optimal control problem:

$$\begin{aligned} \min \quad & J = \frac{1-\alpha}{2} \sum_{k=0}^{N-1} ||C\boldsymbol{x}(t_f) - \boldsymbol{y}_f||_2^2 + \frac{\alpha}{2} \int_0^{t_f} ||\boldsymbol{u}(t)||_2^2 dt \quad && \alpha \in (0,1) \\ \text{s.t.} \quad & \dot{\boldsymbol{x}}_k(t) = A_k \boldsymbol{x}_k(t) + B\boldsymbol{u}(t) && k = 0, \ldots, N-1 \\ & \boldsymbol{x}_k(0) = \bar{\boldsymbol{x}} \end{aligned} \tag{1}$$

where $A_k \in \mathbb{R}^{n \times n}$ $k = 1, 2, \ldots, N$ are square matrices, $B \in \mathbb{R}^{n \times m}$ and $C \in \mathbb{R}^{p \times n}$. Note that while in the main text we impose that B and C have elementary vectors as columns and rows, respectively, the following derivation holds for arbitrary choices of B and C. To solve this problem, we use Pontryagin's minimum principle. The Hamiltonian of Eq. (1) is written as follows by introducing the costates $\boldsymbol{\lambda}_k(t)$, $k = 0, \ldots, N-1$,

$$H(\boldsymbol{x}, \boldsymbol{\lambda}, \boldsymbol{u}) = \frac{\alpha}{2} \boldsymbol{u}^T \boldsymbol{u} + \sum_{k=0}^{N-1} \boldsymbol{\lambda}_k^T A_k \boldsymbol{x}_k + \boldsymbol{\lambda}_k^T B \boldsymbol{u} \tag{2}$$

The evolution of each of the costates, $\boldsymbol{\lambda}_k$, is found by differentiating the Hamiltonian in Eq. (2) with respect to each of the corresponding states $\boldsymbol{x}_k$.

$$\dot{\boldsymbol{\lambda}}_k(t) = -\frac{\partial H}{\partial \boldsymbol{x}_k} = -A_k^T \boldsymbol{\lambda}_k(t) \quad \Rightarrow \quad \boldsymbol{\lambda}_k(t) = e^{A_k^T (t_f - t)} \boldsymbol{\lambda}_k(t_f) \tag{3}$$

To determine the value of the costates at the final time, $t = t_f$, we employ the transversality condition that states,

$$\left[\frac{\partial}{\partial \boldsymbol{x}_k(t_f)} \left(\frac{1-\alpha}{2} ||C\boldsymbol{x}_k(t_f) - \boldsymbol{y}_f||_2^2 \right) - \boldsymbol{\lambda}_k(t_f) \right]^T \delta \boldsymbol{x}_k(t_f) = 0 \tag{4}$$

where $\delta \boldsymbol{x}_k(t_f)$ is a variation on the final values of the states. For Eq. (4) to be satisfied for any value of $\delta \boldsymbol{x}_k(t_f)$, it must be true that,

$$\boldsymbol{\lambda}_k(t_f) = (1-\alpha) C^T (C\boldsymbol{x}_k(t_f) - \boldsymbol{y}_f). \tag{5}$$

Applying this result for the final values of the costates in Eq. (5) to the evolution of the costates in Eq. (4) results in,

$$\boldsymbol{\lambda}_k(t) = (1-\alpha)e^{A_k^T(t_f-t)}C^T(C\boldsymbol{x}_k(t_f) - \boldsymbol{y}_f). \tag{6}$$

The optimal control that minimizes Eq. (1) is found from the stationarity condition, determined by differentiating the Hamiltonian in Eq. (2) with respect to $\boldsymbol{u}$ and setting the result equal to zero.

$$\frac{\partial H}{\partial \boldsymbol{u}} = \alpha \boldsymbol{u}(t) + B^T \sum_{k=0}^{N-1} \boldsymbol{\lambda}_k(t) = \boldsymbol{0}_{n_d} \tag{7}$$

Rearranging Eq. (7) for the optimal control input and applying the result of the time evolution of the costates in Eq. (6),

$$\begin{aligned}\boldsymbol{u}(t) &= -\frac{1}{\alpha}B^T \sum_{k=0}^{N-1} \boldsymbol{\lambda}_k(t) \\ &= -\frac{1-\alpha}{\alpha}B^T \sum_{k=0}^{N-1} e^{A_k^T(t_f-t)}C^T(C\boldsymbol{x}_k(t_f) - \boldsymbol{y}_f)\end{aligned} \tag{8}$$

The original optimal control problem in Eq. (1) has now been transformed into a system of linear equations as the only unknowns are the values of the states at the final time, $\boldsymbol{x}_k(t_f)$. To do this, we solve for the evolution of the states and apply the optimal input found in Eq. (8).

$$\begin{aligned}\boldsymbol{x}_j(t) &= e^{A_j t}\bar{\boldsymbol{x}} - \frac{1-\alpha}{\alpha}\int_0^t e^{A_j\tau}BB^T \sum_{k=0}^{N-1} e^{A_k^T\tau}d\tau e^{A_j^T(t_f-t)}C^T(C\boldsymbol{x}_k(t_f) - \boldsymbol{y}_f) \\ &= e^{A_j t}\bar{\boldsymbol{x}} - \frac{1-\alpha}{\alpha}\sum_{k=0}^{N-1} W_{j,k}(t)e^{A_k^T(t_f-t)}C^T(C\boldsymbol{x}_k(t_f) - \boldsymbol{y}_f)\end{aligned} \tag{9}$$

where the matrix $W_{j,k}(t)$ is the solution of the differential Sylvester equation,

$$\dot{W}_{j,k}(t) = A_j W_{j,k}(t) + W_{j,k}(t)A_k^T + BB^T, \tag{10}$$

which has the formal solution,

$$W_{j,k}(t) = \int_0^t e^{A_j\tau}BB^T e^{A_k^T\tau}d\tau. \tag{11}$$

Note that the blocks are index symmetric, that is, $W_{j,k}(t_f) = W_{k,j}^T(t_f)$. The optimization problem in terms of the unknowns $\boldsymbol{x}_k(t_f)$ can be re-formulated in terms of a related set of variables so that its solution can be found as the solution of a linear system of equations. We call the new variables the *accuracy* of the final state, defined as,

$$\boldsymbol{\gamma}_j(t_f) = C\boldsymbol{x}_j(t_f) - \boldsymbol{y}_f, \quad j = 0, \ldots, N-1 \tag{12}$$

Also, for notational simplicity, we define the *control maneuver* as,

$$\boldsymbol{\beta}_j(t_f) = e^{A_j t_f}\bar{\boldsymbol{x}} - \boldsymbol{y}_f, \quad j = 0, \ldots, N-1 \tag{13}$$

The linear system of equations is constructed by pre-multiplying Eq. (9) by C, setting $t = t_f$, and subtracting $\boldsymbol{y}_f$ from both sides,

$$\alpha\boldsymbol{\beta}_j(t_f) = \alpha\boldsymbol{\gamma}_j(t_f) + (1-\alpha)\sum_{k=0}^{N-1} CW_{j,k}(t_f)C^T\boldsymbol{\gamma}_k(t_f) \tag{14}$$

The global accuracy vectors and control maneuver are of length Np and are defined using Eqs. (12) and (13) as,

$$\begin{aligned}\boldsymbol{\beta}(t_f) &= \begin{bmatrix} \boldsymbol{\beta}_0^T(t_f) & \cdots & \boldsymbol{\beta}_{N-1}^T(t_f) \end{bmatrix}^T \\ \boldsymbol{\gamma}(t_f) &= \begin{bmatrix} \boldsymbol{\gamma}_0^T(t_f) & \cdots & \boldsymbol{\gamma}_{N-1}^T(t_f) \end{bmatrix}^T\end{aligned} \tag{15}$$

The *composite output controllability Gramian* is a symmetric Np-by-Np positive semi-definite matrix defined in terms of N^2 p-by-p blocks $CW_{j,k}(t_f)C^T$,

$$\bar{W}(t_f) = \begin{bmatrix} CW_{0,0}(t_f)C^T & CW_{0,1}(t_f)C^T & \cdots & CW_{0,N-1}(t_f)C^T \\ CW_{1,0}(t_f)C^T & CW_{1,1}(t_f)C^T & \cdots & CW_{1,N-1}(t_f)C^T \\ \vdots & \vdots & \ddots & \vdots \\ CW_{N-1,0}(t_f)C^T & CW_{N-1,1}(t_f)C^T & \cdots & CW_{N-1,N-1}(t_f)C^T \end{bmatrix} \tag{16}$$

where each matrix $W_{j,k}(t_f)$ is defined in Eqs. (10) and (11). The optimal accuracy vector $\boldsymbol{\gamma}(t_f)$ can be determined by using the definitions in Eqs. (15) and (16) in Eq. (14),

$$\left(\alpha I_{Np} + (1-\alpha)\bar{W}(t_f)\right)\boldsymbol{\gamma}(t_f) = \bar{U}(\alpha)\boldsymbol{\gamma}(t_f) = \alpha\boldsymbol{\beta}(t_f) \tag{17}$$

Once the accuracy vectors are known, $\boldsymbol{\gamma}_k(t_f) = C\boldsymbol{x}_k(t_f) - \boldsymbol{y}_f$, $k = 0, \ldots, N-1$, they can be used to evaluate the optimal control input in Eq. (8) or the optimal values of the states in Eq. (9).

Quadratic Forms of the Costs

The optimal control energy and the optimal deviation can be expressed as quadratic forms using Eq. (17) and the optimal input in Eq. (8). The control energy can be rewritten as

$$\begin{aligned} E_N(\alpha) &= \int_0^{t_f} \boldsymbol{u}^T(t)\boldsymbol{u}(t)dt \\ &= \frac{(1-\alpha)^2}{\alpha^2}\sum_{j,k=0}^{N-1}\boldsymbol{\gamma}_j^T(t_f)C\left[\int_0^{t_f} e^{A_j(t_f-t)}BB^Te^{A_k^T(t_f-t)}dt\right]C^T\boldsymbol{\gamma}_k(t_f) \\ &= \frac{(1-\alpha)^2}{\alpha^2}\boldsymbol{\gamma}^T(t_f)\bar{W}\boldsymbol{\gamma}(t_f) \\ &= (1-\alpha)^2\boldsymbol{\beta}^T(t_f)\bar{U}^{-1}(\alpha)\bar{W}\bar{U}^{-1}(\alpha)\boldsymbol{\beta}(t_f) \end{aligned} \tag{18}$$

and the deviation can be rewritten,

$$
\begin{aligned}
D_N(\alpha) &= \sum_{k=0}^{N-1} \boldsymbol{\gamma}_k^T(t_f)\boldsymbol{\gamma}_k(t_f) \\
&= \boldsymbol{\gamma}^T(t_f)\boldsymbol{\gamma} \\
&= \alpha^2 \boldsymbol{\beta}^T(t_f)\bar{U}^{-1}(\alpha)\bar{U}^{-1}(\alpha)\boldsymbol{\beta}(t_f)
\end{aligned} \tag{19}
$$

The quadratic forms in Eqs. (18) and (19) can be expressed in terms of the spectrum of the composite output controllability Gramian. Combining Eqs. (18) and (19) we see that the total cost can also be expressed as a quadratic form,

$$
\begin{aligned}
J_N(\alpha) &= \frac{1-\alpha}{2} D_N(\alpha) + \frac{\alpha}{2} E_N(\alpha) \\
&= \frac{(1-\alpha)\alpha}{2} \boldsymbol{\beta}^T(t_f)\bar{U}^{-1}(\alpha)\left(\alpha I_{Np} + (1-\alpha)\bar{W}(t_f)\right)\bar{U}^{-1}(\alpha)\boldsymbol{\beta}(t_f) \\
&= \frac{(1-\alpha)\alpha}{2} \boldsymbol{\beta}^T(t_f)\bar{U}^{-1}(\alpha)\boldsymbol{\beta}(t_f)
\end{aligned} \tag{20}
$$

Summation Forms of the Costs

In the following derivations, the eigenvalues and eigenvectors of the COCG in Eq. (16) will play an important role. The eigenvalues of $W(t_f)$ are denoted as μ_k, ordered such that $\mu_k \geq \mu_{k+1}$, with corresponding eigenvectors $\boldsymbol{\xi}_k$ so that $\bar{W}(t_f)\boldsymbol{\xi}_k = \mu_k \boldsymbol{\xi}_k$. Note that the matrices $\bar{W}(t_f)$ and $\bar{U}(\alpha)$ are *simultaneously diagonalizable*, that is, they share their eigenbasis, but for each eigenvalue of $\bar{W}(t_f)$, μ_k, there is a corresponding eigenvalue of $\bar{U}(\alpha)$, denoted ν_k,

$$
\nu_k = \alpha + (1-\alpha)\mu_k \tag{21}
$$

Let Ξ be the Np-by-Np matrix with the eigenvectors $\boldsymbol{\xi}_j$, $j = 0, \ldots, Np-1$, as columns. Note that as $\bar{W}(t_f)$ is a symmetric matrix, its eigenbasis is *orthonormal* so that $\Xi\Xi^T = I_{Np}$. Also, define the Np-by-Np diagonal matrices $\mathcal{M}$ and $\mathcal{N}$ as having the eigenvalues μ_j and ν_j along their diagonals, respectively. Finally, the projection of the control maneuver into the eigenbasis is written as $\boldsymbol{\theta} = \Xi^T \boldsymbol{\beta}(t_f)$. Using the notation for the spectral properties of $\bar{W}(t_f)$ defined above, the control energy in Eq. (18) can equivalently be written as a summation.

$$
\begin{aligned}
E_N(\alpha) &= (1-\alpha)^2 \boldsymbol{\beta}^T(t_f)\Xi\Xi^T\bar{U}^{-1}\Xi\Xi^T\bar{W}(t_f)\Xi\Xi^T\bar{U}^{-1}(\alpha)\Xi\Xi^T\boldsymbol{\beta}(t_f) \\
&= (1-\alpha)^2 \boldsymbol{\theta}^T \mathcal{N}^{-1}\mathcal{M}\mathcal{N}^{-1}\boldsymbol{\theta} \\
&= (1-\alpha)^2 \sum_{k=0}^{Np-1} \frac{\mu_k \theta_k^2}{(\alpha + (1-\alpha)\mu_k)^2}
\end{aligned} \tag{22}
$$

In a similar manner, the deviation in Eq. (19) can be expressed as a summation.

$$
\begin{aligned}
D_N(\alpha) &= \alpha^2 \boldsymbol{\beta}^T(t_f)\Xi\Xi^T\bar{U}^{-1}(\alpha)\Xi\Xi^T\bar{U}^{-1}(\alpha)\Xi\Xi^T\boldsymbol{\beta}(t_f) \\
&= \alpha^2 \boldsymbol{\theta}^T\mathcal{N}^{-1}\mathcal{N}^{-1}\boldsymbol{\theta}\alpha^2 \sum_{k=0}^{Np-1} \frac{\theta_k^2}{(\alpha + (1-\alpha)\mu_k)^2}
\end{aligned} \tag{23}
$$

The optimal cost is also expressed as a summation.

$$\begin{aligned} J_N(\alpha) &= \frac{(1-\alpha)\alpha}{2}\boldsymbol{\beta}^T(t_f)\Xi\Xi^T\bar{U}^{-1}(\alpha)\Xi\Xi^T\boldsymbol{\beta}(t_f) \\ &= \frac{(1-\alpha)\alpha}{2}\boldsymbol{\theta}^T\mathcal{N}^{-1}\boldsymbol{\theta} \\ &= \frac{(1-\alpha)\alpha}{2}\sum_{k=0}^{Np-1}\frac{\theta_k^2}{\alpha+(1-\alpha)\mu_k} \end{aligned} \tag{24}$$

These three expressions are used in the following sections where we apply the approximate behavior of the eigenvalues, μ_k, and squared projection of the control maneuver on the eigenvectors, θ_k^2.

Approximation of the Costs

In the main text, the following two assumptions are presented related to μ_k and θ_k^2 which appear to hold numerically.

$$\begin{aligned} &\textbf{Assumption 1:} \quad \mu_k \approx \mu_0 r_1^k, && \mu_0 \approx c_1 Np \\ &\textbf{Assumption 2:} \quad \theta_k^2 \approx \max\{\theta_0^2 r_2^k, \theta_c^2\}, && \theta_0 \approx c_2 Np \end{aligned} \tag{25}$$

The most important index is the switching index when θ_k^2 stops decaying exponentially and becomes constant. To determine this index, $\bar{k}$, we solve the following inequality in terms of the largest index k for which it holds true.

$$c_2 Np r_2^k \geq \theta_c^2 \quad \Rightarrow \quad \bar{k} = \max\left\{\left\lfloor\frac{\log(\theta_c^2)-\log(c_2 Np)}{\log r_2}\right\rfloor, 0\right\} \tag{26}$$

Note that as $r_2 \in (0,1)$ the denominator is strictly negative. To ensure $\bar{k} \geq 0$, we choose $Np \geq \frac{\theta_c^2}{c_2}$ so that the numerator is also negative. Applying Assumption 1 and Assumption 2 to the summation form of the control energy in Eq. (22), the deviation in Eq. (23), and the optimal cost in Eq. (24), lead to the following approximate forms,

$$\begin{aligned} E_N(\alpha) \approx \bar{E}_N(\alpha) &= (1-\alpha)^2 c_1 c_2 (Np)^2 \sum_{k=0}^{\bar{k}} \frac{r_1^k r_2^k}{(\alpha+(1-\alpha)c_1 Np r_1^k)^2} \\ &+ (1-\alpha)^2 c_1 Np \theta_c^2 \sum_{k=\bar{k}+1}^{Np-1} \frac{r_1^k}{(\alpha+(1-\alpha)c_1 Np r_1^k)^2} \end{aligned} \tag{27}$$

The control energy approximation can be bounded from above and below which we call,

$$\bar{E}_{N,LB}(\alpha) \leq \bar{E}_N(\alpha) \leq \bar{E}_{N,UB}(\alpha) \tag{28}$$

The upper bound is determined by taking only the dominant term in the denominator of the summations.

$$\bar{E}_N(\alpha) = c_1 c_2 \sum_{k=0}^{\bar{k}} \frac{(r_1 r_2)^k}{\left(\frac{\alpha}{(1-\alpha)Np} + c_1 r_1^k\right)^2} + \frac{c_1\theta_c^2}{Np}\sum_{k=\bar{k}+1}^{Np-1}\frac{r_1^k}{\left(\frac{\alpha}{(1-\alpha)Np} + c_1 r_1^k\right)^2} \tag{29}$$

With this rearrangement, the upper bound is,

$$\begin{aligned}\bar{E}_{N,UB}(\alpha) &= c_1c_2\left(\frac{(1-\alpha)Np}{\alpha}\right)^2\sum_{k=0}^{\bar{k}}(r_1r_2)^k + \frac{c_1\theta_c^2}{Np}\left(\frac{(1-\alpha)Np}{\alpha}\right)^2\sum_{k=\bar{k}+1}^{Np-1}r_1^k \\ &= \left[c_1c_2\left(\frac{1-(r_1r_2)^{\bar{k}+1}}{1-r_1r_2}\right)+\frac{c_1\theta_c^2}{Np}\left(\frac{r_1^{\bar{k}+1}-r_1^{Np}}{1-r_1}\right)\right]\left(\frac{(1-\alpha)Np}{\alpha}\right)^2\end{aligned} \tag{30}$$

The lower bound can be determined by first taking only the decaying portion of Assumption 2,

$$\begin{aligned}\bar{E}_N(\alpha) &\geq c_1c_2\sum_{k=0}^{Np-1}\frac{(r_1r_2)^k}{\left(\frac{\alpha}{(1-\alpha)Np}+c_1r_1^k\right)^2} \\ &\geq c_1c_2\left(\frac{\alpha}{(1-\alpha)Np}+c_1\right)^{-2}\sum_{k=0}^{Np-1}(r_1r_2)^k \\ &= c_1c_2\left(\frac{\alpha}{(1-\alpha)Np}+c_1\right)^{-2}\left(\frac{1-(r_1r_2)^{Np}}{1-r_1r_2}\right)\end{aligned} \tag{31}$$

The upper and lower bounds depend on the important quantity $\frac{(1-\alpha)Np}{\alpha}$ which will be used to fix the bounds in the next section.

The deviation approximation is first written using the Assumptions.

$$\begin{aligned}D_N(\alpha) \approx \bar{D}_N(\alpha) &= \alpha^2c_2Np\sum_{k=0}^{\bar{k}}\frac{r_2^k}{(\alpha+(1-\alpha)c_1Npr_1^k)^2} \\ &+ \alpha^2\theta_c^2\sum_{k=\bar{k}+1}^{Np-1}\frac{1}{(\alpha+(1-\alpha)c_1Npr_1^k)^2}\end{aligned} \tag{32}$$

Performing the same shifting of α to appear in a single term in the denominator and dividing by the number of realizations,

$$\begin{aligned}\frac{\bar{D}_N(\alpha)}{Np} &= c_2\left(\frac{\alpha}{(1-\alpha)Np}\right)^2\sum_{k=0}^{\bar{k}}\frac{r_2^k}{\left(\frac{\alpha}{(1-\alpha)Np}+c_1r_1^k\right)^2} \\ &+ \left(\frac{\alpha}{(1-\alpha)Np}\right)^2\frac{\theta_c^2}{Np}\sum_{k=\bar{k}+1}^{Np-1}\frac{1}{\left(\frac{\alpha}{(1-\alpha)Np}+c_1r_1^k\right)^2}\end{aligned} \tag{33}$$

An upper bound for the average deviation can be determined by taking only the α dependent term in the denominators,

$$\frac{\bar{D}_N(\alpha)}{Np} \leq \frac{\bar{D}_{N,UB}(\alpha)}{Np} = c_2\sum_{k=0}^{\bar{k}}r_2^k + \frac{\theta_c^2}{Np}\sum_{k=0}^{Np-1}1 = c_2\left(\frac{1-r_2^{\bar{k}+1}}{1-r_2}\right)+\theta_c^2 \tag{34}$$

which is, importantly, independent of α, which demonstrates that the average deviation remains finite regardless of the choice of N or α. Upon reflection though, this result is

expected given our assumptions of either finite time, t_f, or if $t_f \to \infty$, then every realization A_j being Hurwitz, $j = 1, \ldots, N$.
For completeness, we also include the approximate form of the total cost so that we may evaluate it when we impose a particular form for α.

$$
\begin{aligned}
J_N(\alpha) \approx \bar{J}_N(\alpha) = \frac{(1-\alpha)\alpha}{2} c_2 Np \sum_{k=0}^{\bar{k}} \frac{r_2^k}{\alpha + (1-\alpha)c_1 Np r_1^k} \\
+ \frac{(1-\alpha)\alpha}{2} \theta_c^2 \sum_{k=\bar{k}+1}^{Np-1} \frac{1}{\alpha + (1-\alpha)c_1 Np r_1^k}
\end{aligned}
\tag{35}
$$

In the next section, we will show that, while the average deviation remains finite regardless of the choice of α, an improper selection can lead to diverging control energy, $E_N(\alpha)$. We will also show though that a proper choice of $\alpha(N)$, that is, treating the weighting parameter as a function of N, can be used to introduce a more useful parameter that can tune the approximate average deviation to any desired positive value while also ensuring the control energy will not diverge in the thermodynamic limit.

Proper Choice of α

So far, the parameter α has been assumed to be independent of N. This choice does not take into account the fact that the number of terms in the definition of $D_N(\alpha)$ grows with N. As a result, the behavior of $D_N(\alpha)/Np$ and $E_N(\alpha)$ as a function of N for fixed α is often surprising and may lead to misleading conclusions.

Instead, we impose the following form for $\alpha = \alpha(N)$,

$$
\alpha(N) = \frac{Np}{Np + b}, \quad b > 0 \tag{36}
$$

Applying this choice to the upper and lower bounds of the control energy derived previously in Eq. (30) and Eq. (31),

$$
\bar{E}_{N,UB}(b) = b^2 \left[c_1 c_2 \left(\frac{1 - (r_1 r_2)^{\bar{k}+1}}{1 - r_1 r_2} \right) + \frac{c_1 \theta_c^2}{Np} \left(\frac{r_1^{\bar{k}+1} - r_1^{Np}}{1 - r_1} \right) \right] \tag{37}
$$

$$
\bar{E}_{N,LB}(b) = c_1 c_2 \left(\frac{b}{1 + c_1 b} \right)^2 \left(\frac{1 - (r_1 r_2)^{Np}}{1 - r_1 r_2} \right) \tag{38}
$$

where we used the expression,

$$
\frac{(1-\alpha)Np}{\alpha} = b \tag{39}
$$

which, unlike in the previous case for constant α, converges in the thermodynamic limit,

$$
\lim_{N \to \infty} \bar{E}_{N,UB}(b) = b^2 \left[\frac{c_1 c_2}{1 - r_1 r_2} \right] \tag{40}
$$

Note that the upper bound of the control energy approximation asymptotically grows quadratically with b.

Moving to the average deviation, we have already shown that it is upper bounded regardless of the choice of $\alpha(N)$. What remains though is to show that given our choice of $\alpha(N)$ in Eq. (36) there exists a value of b to tune the upper bound of the average deviation to any value desired. To do this, we show that the average deviation $\bar{D}_N(b)/Np$ is a bijection from $[0, \infty) \to (0, \bar{D}(0)/Nb]$ by showing that,

1. $\lim_{b\to\infty} \frac{\bar{D}_N(b)}{Np} = 0$ and

2. $\frac{d}{db}\frac{\bar{D}_N(b)}{Np} < 0$

First, we apply our choice of $\alpha(N)$ to Eq. (33),

$$\frac{\bar{D}_N(b)}{Np} = c_2 \sum_{k=0}^{\bar{k}} \frac{r_2^k}{(1+bc_1 r_1^k)^2} + \frac{\theta_c^2}{Np} \sum_{k=\bar{k}+1}^{Np-1} \frac{1}{(1+bc_1 r_1^k)^2} \tag{41}$$

Holding the value of N constant, we may take the limit of large b.

$$\lim_{b\to\infty} \frac{\bar{D}_N(b)}{Np} = 0 \tag{42}$$

This implies that the average deviation, for our choice of $\alpha(N)$, remains bounded between,

$$0 < \frac{\bar{D}_N(b)}{Np} < c_2 \left(\frac{1-r_2^{\bar{k}}}{1-r_2}\right) + \theta_c^2, \quad b \in (0, \infty) \tag{43}$$

To show that there exists a value of b to set the upper bound of the average deviation to any desired value below the upper bound, it suffices to show that the derivative of the average deviation with respect to b is strictly negative, i.e., the average deviation is a monotonically decreasing function.

$$\frac{\partial}{\partial b}\frac{\bar{D}_N(b)}{Np} = -2c_1c_2 \sum_{k=0}^{\bar{k}} \frac{(r_1 r_2)^k}{(1+bc_1 r_1^k)^3} - \frac{2c_1\theta_c^2}{Np} \sum_{k=\bar{k}+1}^{Np-1} \frac{r_1^k}{(1+bc_1 r_1^k)^3} < 0 \tag{44}$$

We have thus proven that the average deviation can be reduced by increasing b with the trade-off that the approximate control energy grows exponentially.

Finally, the optimal cost with the assumptions in Eq. (25) and our choice of $\alpha(N)$ in Eq. (36) applied becomes approximately

$$J_N(b) \approx \bar{J}_N(b) = \frac{bNp}{2(Np+b)} c_2 \sum_{k=0}^{\bar{k}} \frac{r_2^k}{\alpha + bc_1 r^k} + \frac{b}{2(Np+b)} \theta_c^2 \sum_{k=\bar{k}+1}^{Np-1} \frac{1}{1+bc_1 r^k} \tag{45}$$

The approximate total cost is upper bounded using the same technique as was used for the control energy and average deviation above.

$$\bar{J}_N(b) < \frac{bNp}{2(Np+b)} c_2 \sum_{k=0}^{\bar{k}} r_2^k + \frac{b\theta_c^2}{2(Np+b)} \sum_{k=0}^{Np-1} 1 = \frac{bNpc_2}{2(Np+b)} \frac{1-r_2^{\bar{k}+1}}{1-r_2} + \frac{b\theta_c^2}{2(Np+b)} Np \tag{46}$$

Holding N constant, at the two bounds $b = 0$ and $b \to \infty$, the total cost is zero. For intermediate values of b though, the total cost $\bar{J}_N(b) > 0$, and achieves at least one local maxima.

Alternative Method to Derive Proper Choice of $\alpha(N)$

An alternative way to show that a proper choice of α is needed in order to make the solution of the optimal control problem feasible in the large N limit is included below. Let $\bar{u}(t)$ be any integrable sub-optimal control input, and let $\gamma^{\max}$ be the resulting maximum difference of any state realization,

$$pd^{\max} = \max_{A^{(k)} \in \mathcal{A}} \left|\left| y_f - C \left(e^{A^{(k)}(t_f - t_0)} x_0 + \int_{t_0}^{t_f} e^{A^{(k)}(t_f - \tau)} B\bar{u}(\tau) d\tau \right) \right|\right|_2^2 \quad (47)$$

The optimal cost can now be upper bounded by,

$$J \leq \bar{J} = \frac{1-\alpha}{2} N d^{\max} + \frac{\alpha}{2} \int_{t_0}^{t_f} ||\bar{u}(t)||_2^2 dt \quad (48)$$

Note that the energy term is a constant independent of N while the first term grows linearly with N. To compensate for this growth, we need $(1-\alpha)$ to decay as $\frac{1}{N}$, so we choose

$$\alpha = \frac{Np}{Np + b} \quad (49)$$

This upper bound can then be expressed as,

$$\bar{J} = \frac{b}{2(Np+b)} Npd^{\max} + \frac{Np}{2(Np+b)} \int_{t_0}^{t_f} ||\bar{u}(t)||_2^2 dt \quad (50)$$

Taking the limit of this expression for constant b yields the asymptotic behavior,

$$\lim_{N \to \infty} \bar{J} = \frac{bd^{\max}}{2} + \frac{1}{2} \int_{t_0}^{t_f} ||\bar{u}(t)||_2^2 dt \quad (51)$$

which is a constant.

Supplementary Note 2: Additional Results Concerning the Uncertain Unidirectional Chain Graph

As an example, we return to the uncertain unidirectional chain system shown in Fig. 3(A) of the main text except this time we choose the set of target nodes to be a single node some distance from the driver nodes, $\mathcal{T} = \{v_k\}$. The same distributions, that is $p_k \in \mathcal{U}(2,4)$ and $s_k \in \mathcal{U}(0.9, 1.1)$, for $k = 0, \dots, N-1$, are used for this simulation. The results deviation for 25 realizations of $N = 100$ for increasing values of b are shown in Supplementary Figure 1(A), the control energy in Supplementary Figure 1(B), and the total cost in Supplementary Figure 1(C). What is most notable is that the deviation curves appear to have a dynamic range, within which the deviation changes rapidly with respect to b and outside of which the deviation changes very little. As k grows, the dynamic range shifts to larger values of b. This can be explained from the result that as k grows linearly, c_1 decays exponentially, leading to the product that appears in the denominators of all of the terms in Eqs. (27), (32) and (35), bc_1, to require larger values of b to result in similar behavior. The control

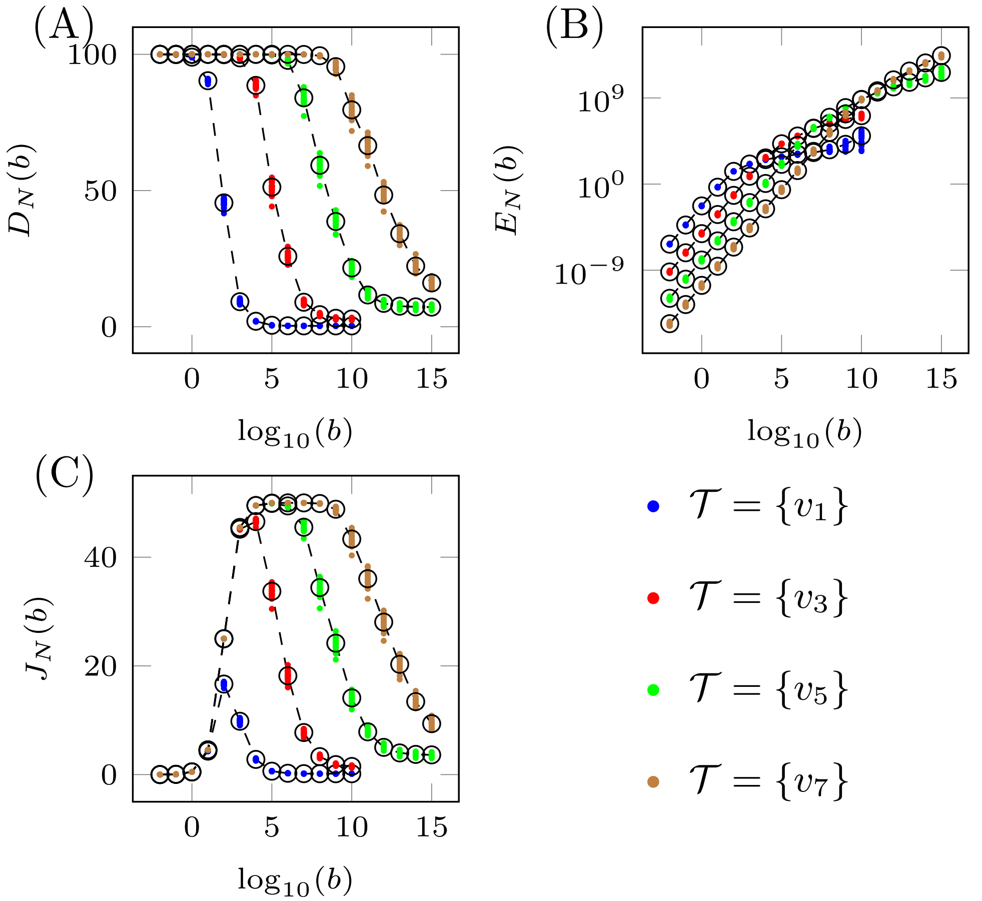

Supplementary Figure 1: The deviation, control energy and total cost as a function of the weighting parameters b. Each colored mark corresponds to a realization of the uncertain unidirectional chain network discussed previously where $N = 100$ and $y_f = 1$, and the black open circle represents the average over 25 realizations. (A) The deviation, which for small b, approaches the maximum value, $\boldsymbol{\beta}^T\boldsymbol{\beta}$, while for large b, approaches zero. (B) The control energy, which for small b approaches zero while for large b approaches the maximum value $\boldsymbol{\beta}^T\bar{W}^{-1}\boldsymbol{\beta}$. (C) The total cost, which is zero for both $b = 0$ and $b = 1$, while it reaches a maximum for some intermediate value.

energy has two clear regimes; for small b we see that the control energy grows at approximately the same rate regardless of the target node while for large b, there is a decrease in the rate of growth of the control energy, but the resulting curves are not growing at the same rate. These energy curves will approach different constants in the limit of $b \to \infty$, equal to $\boldsymbol{\beta}^T \bar{W}^{-1} \boldsymbol{\beta}$. The total cost in Supplementary Figure 1(C) show a maximum value for some value of b, while it decays to 0 in both the $b \to 0$ limit and the $b \to \infty$ limit.
